# Supplementary material for: Evolution in action: habitat transition from sediment to the pelagial leads to genome streamlining in Methylophilaceae
Source: ISME J. 2019 Jul 10;13(11):2764–77. doi: 10.1038/s41396-019-0471-3 (PMC6794327; doi:10.1038/s41396-019-0471-3)
Supplement: Supplementary file 1 — Supplemental Figures [file 41396_2019_471_MOESM1_ESM.pdf]

Supplementary information for

Evolution in action: Habitat-transition from sediment to the pelagial leads to genome-streamlining in Methylophilaceae

Michaela M. Salcher, Daniel Schaefle, Melissa Kaspar, Stefan M. Neuenschwander, Rohit Ghai

Supplementary Tables S1-S5 and a set of HMMs of can be found as separate dataset (SalcherTablesS1-S5.xlsx; Salcher_methdbHMM.gz)

Table S1: Characteristics of analysed genomes.

Table S2: Public metagenomes used for recruitment analyses and RPKG values (reads per kb of genome per Gb of metagenome) of all genomes.

Table S3: Membrane transporters annotated in the genomes.

Table S4: Selected metabolic modules present in the genomes.

Table S5: Complete list of proteins used for creating HMMs related to methylotrophic functions. The entire set of HMMs is available as supplementary data set (Salcher_meth-HMMs.txt)

Figure S1: Phylogeny of 39 strains of '*Ca*. Methylopumilus spp.'.

Figure S2: Average nucleotide identity (ANI) of 39 strains of '*Ca*. Methylopumilus sp.'.

Figure S3: Average amino acid identity (AAI) of Methylophilaceae.

Figure S4: Average nucleotide identity (ANI) of Methylophilaceae.

Figure S5: Genomic statistics of Methylophilaceae.

Figure S6: Amino acid usage of Methylophilaceae in relation to genome size.

Figure S7: Core- and pangenome analysis of Methylophilaceae.

Figure S8: Methylcitric acid (MCA) pathway.

Figure S9: Conserved methylotrophic pathways - H4F, methanol oxidation and RuMP.

Figure S10: Conserved methylotrophic pathways - methanol and formate oxidation.

Figure S11: XoxF and MxaF type methanol dehydrogenases.

Figure S12: Horizontal gene transfers of different rhodopsins.

Figure S13: Horizontal gene transfer of the NQR pathway in marine HTCC2181.

Figure S14: Horizontal gene transfer of an ectoine biosynthesis pathway and a sodium:proline symporter in marine OM43

Figure S15: Horizontal gene transfer an oligopeptide permease in marine OM43

Figure S16: Horizontal gene transfer of sodium transporters in marine OM43

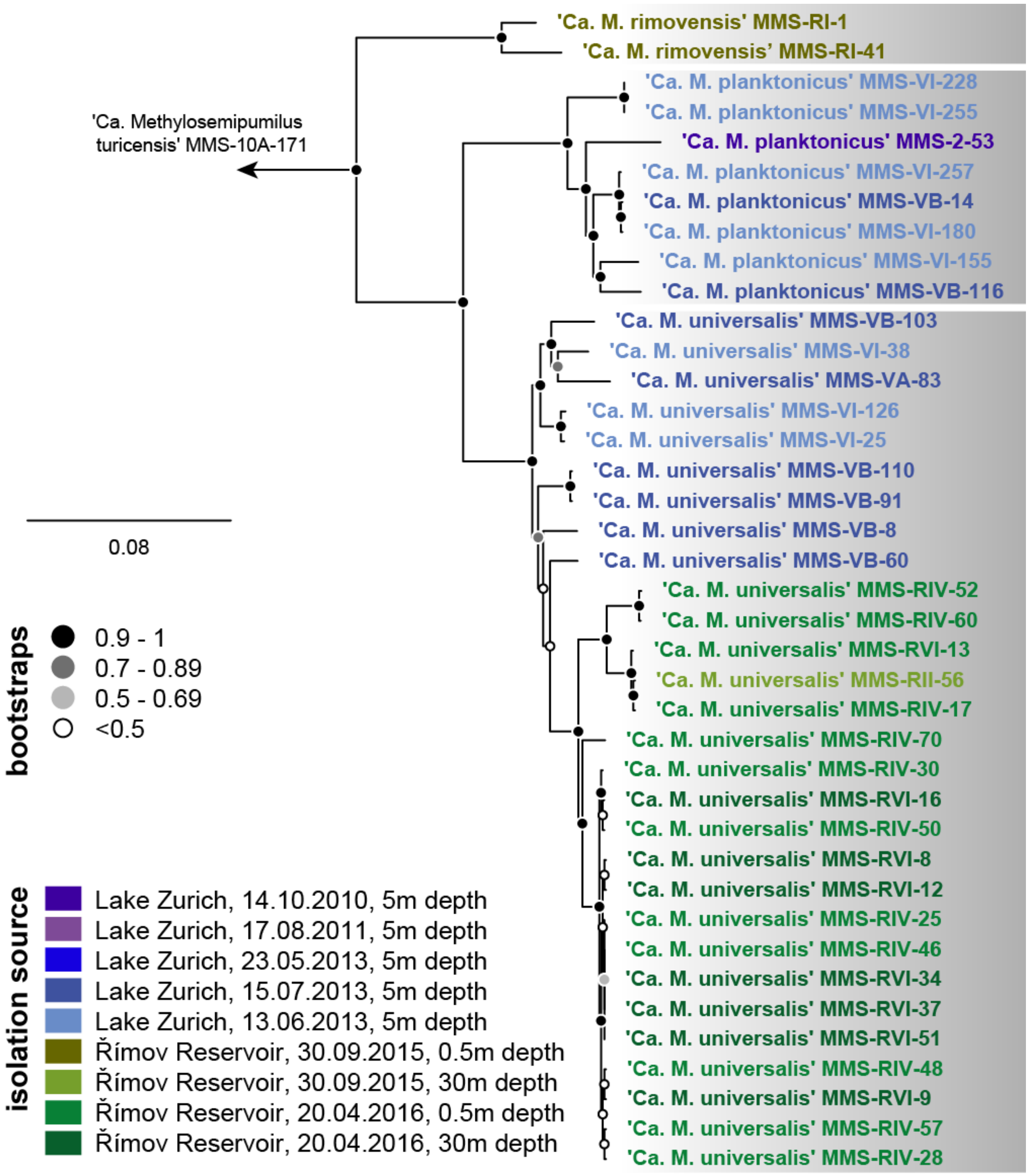

Figure S1: Phylogeny of 39 strains of '*Ca*. Methylopumilus spp.'.

Phylogenomic tree of 39 complete genomes of 'Ca. Methylopumilus spp' that form three distinct species ('Ca. M. rimovensis', 'Ca. M. planktonicus', 'Ca. M. universalis'). The tree is based on 983 common, concatenated protein sequences (337501 amino acid sites) with 'Ca. Methylosemipumilus turicensis' used as outgroup. Bootstraps values (100 bootstraps) are indicated by circles at individual nodes, the scale bar applies to 8% sequence divergence. The isolation source is indicated by different colours.

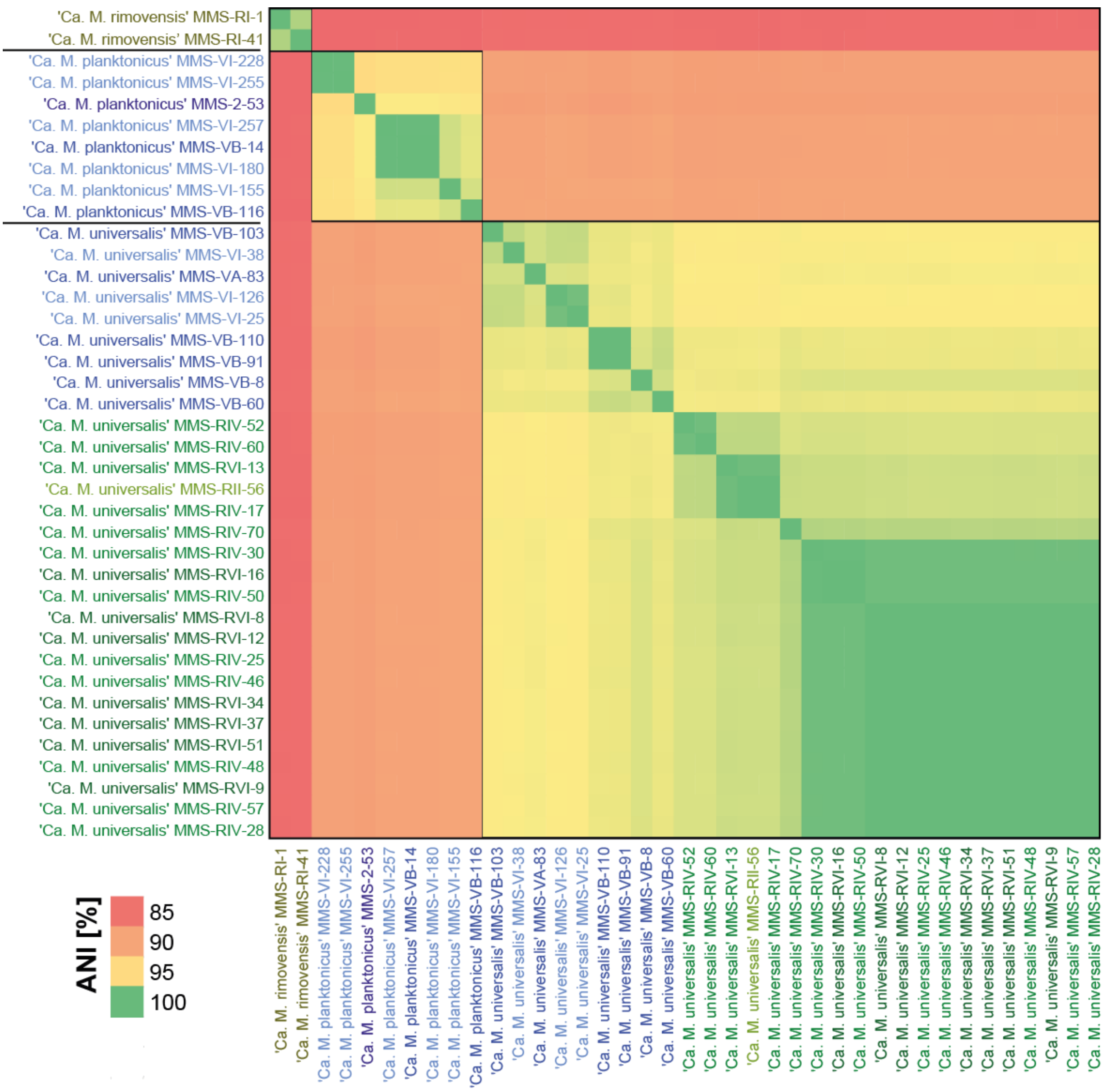

Figure S2: Average nucleotide identity (ANI) of 39 strains of '*Ca*. Methylopumilus sp.'.

Values >95% identity (species level delineation) are marked by black boxes. The colour coding of genomes is according to isolation source as displayed in Fig. S1.

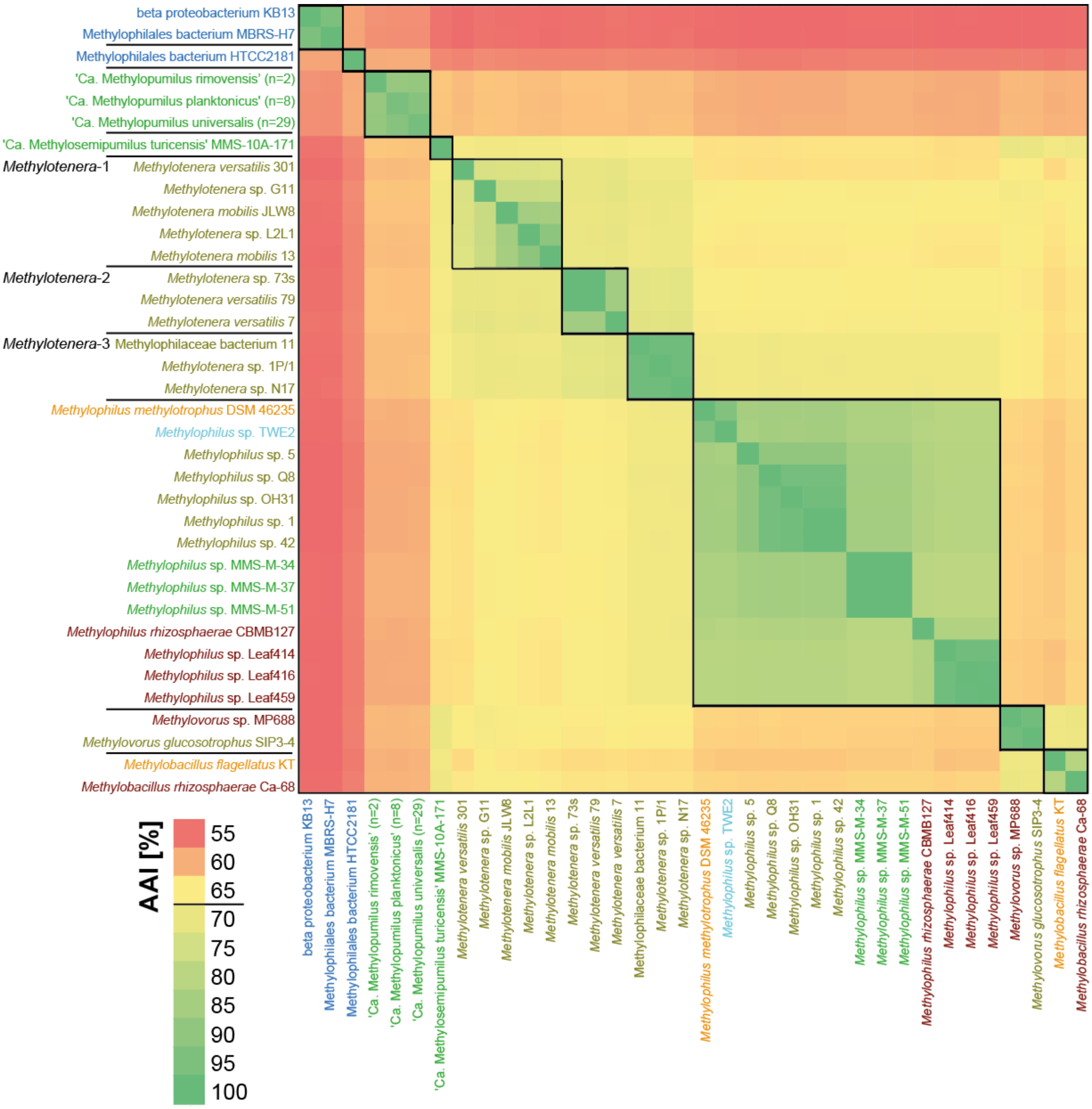

Figure S3: Average amino acid identity (AAI) of Methylophilaceae.

Values >70% identity (approx. genus level delineation) are marked by black boxes. The colour coding of genomes is according to Fig. 1.

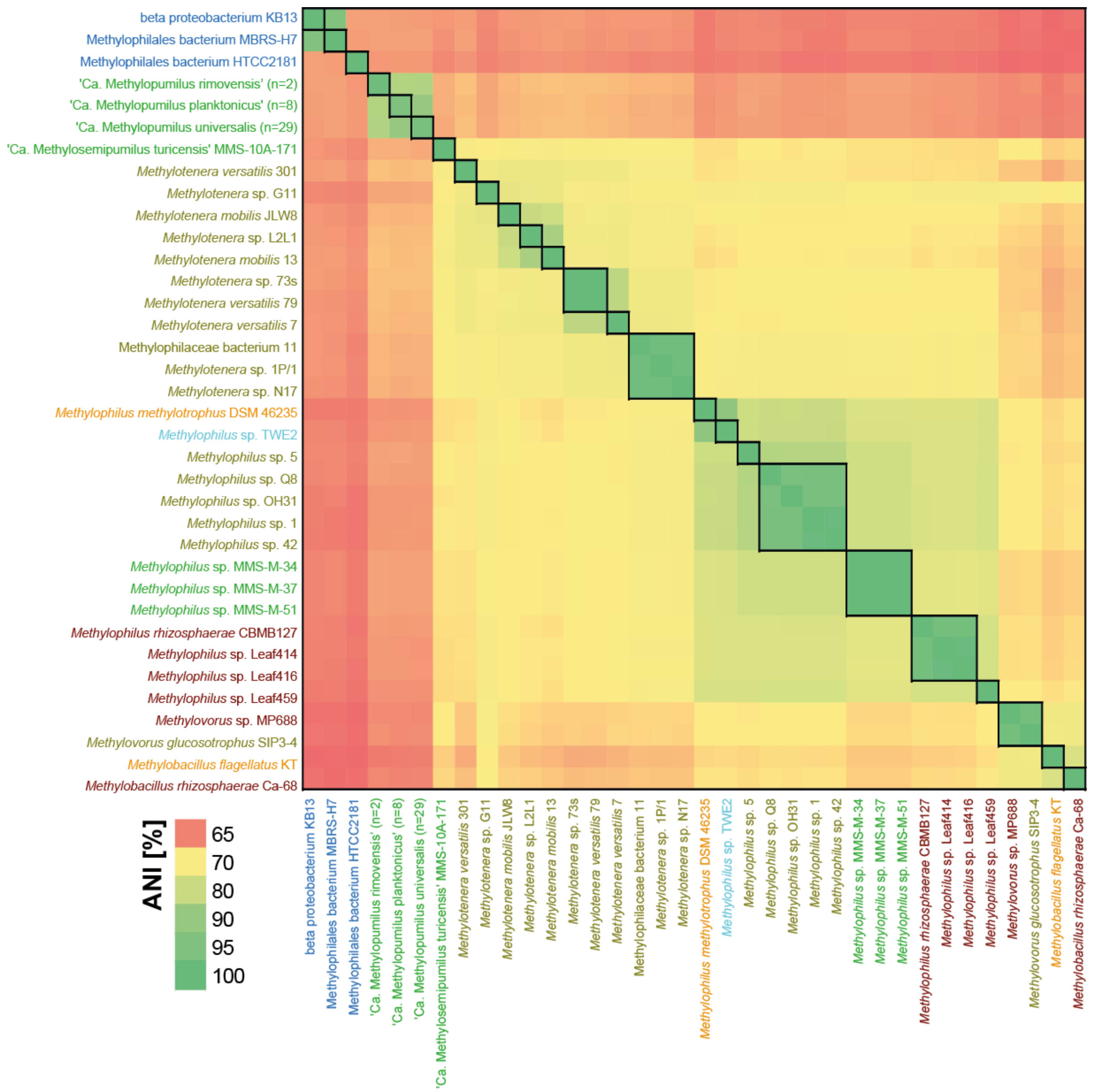

Figure S4: Average nucleotide identity (ANI) of Methylophilaceae.

Values >95% identity (species level delineation) are marked by black boxes. The colour coding of genomes is according to Fig. 1.

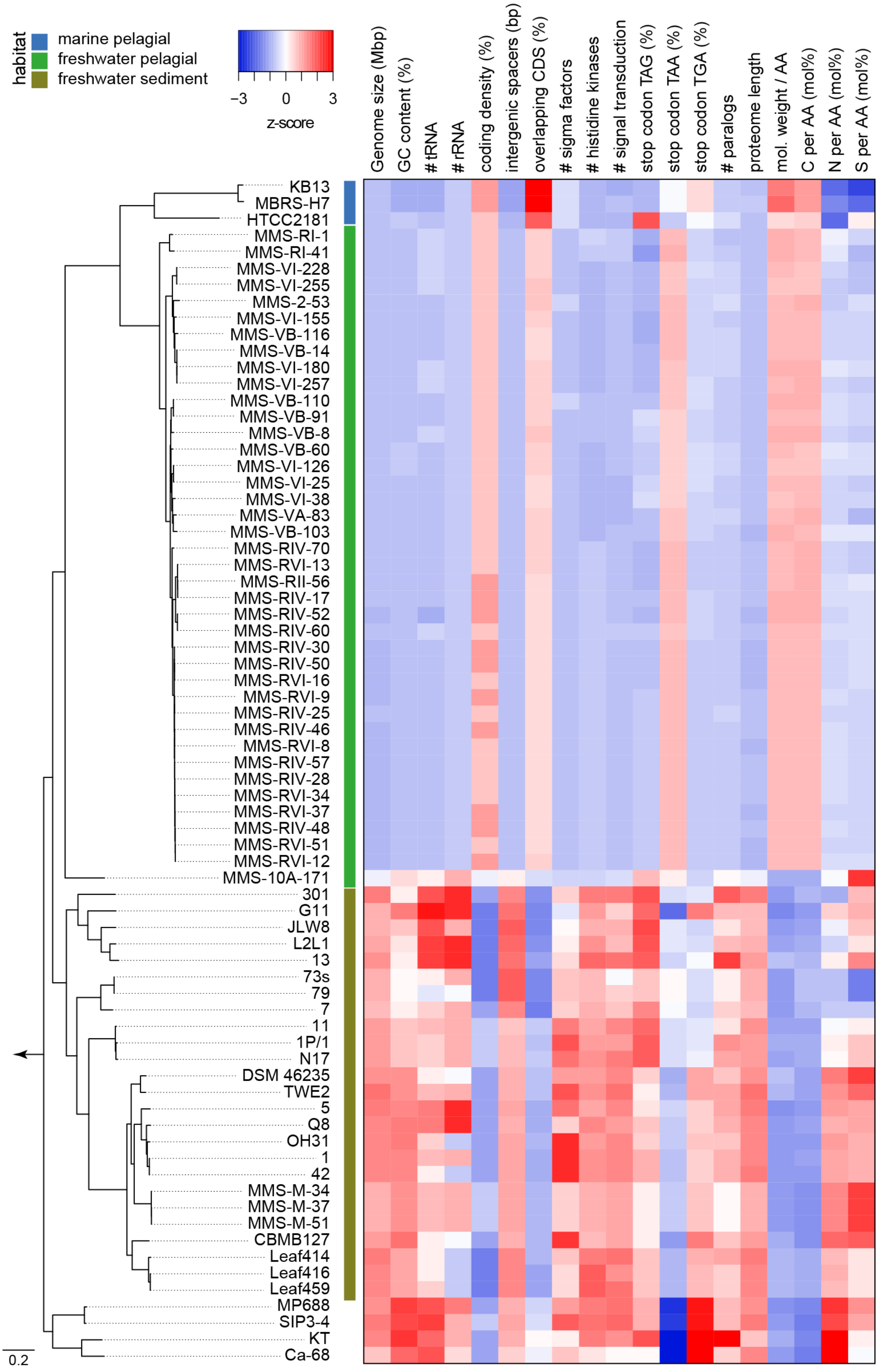

Figure S5: Genomic statistics of Methylophilaceae.

Phylogenomic tree (for details see Fig. 1 and Fig. S1) with selected genomic features related to genome-streamlining in Methylophilaceae.

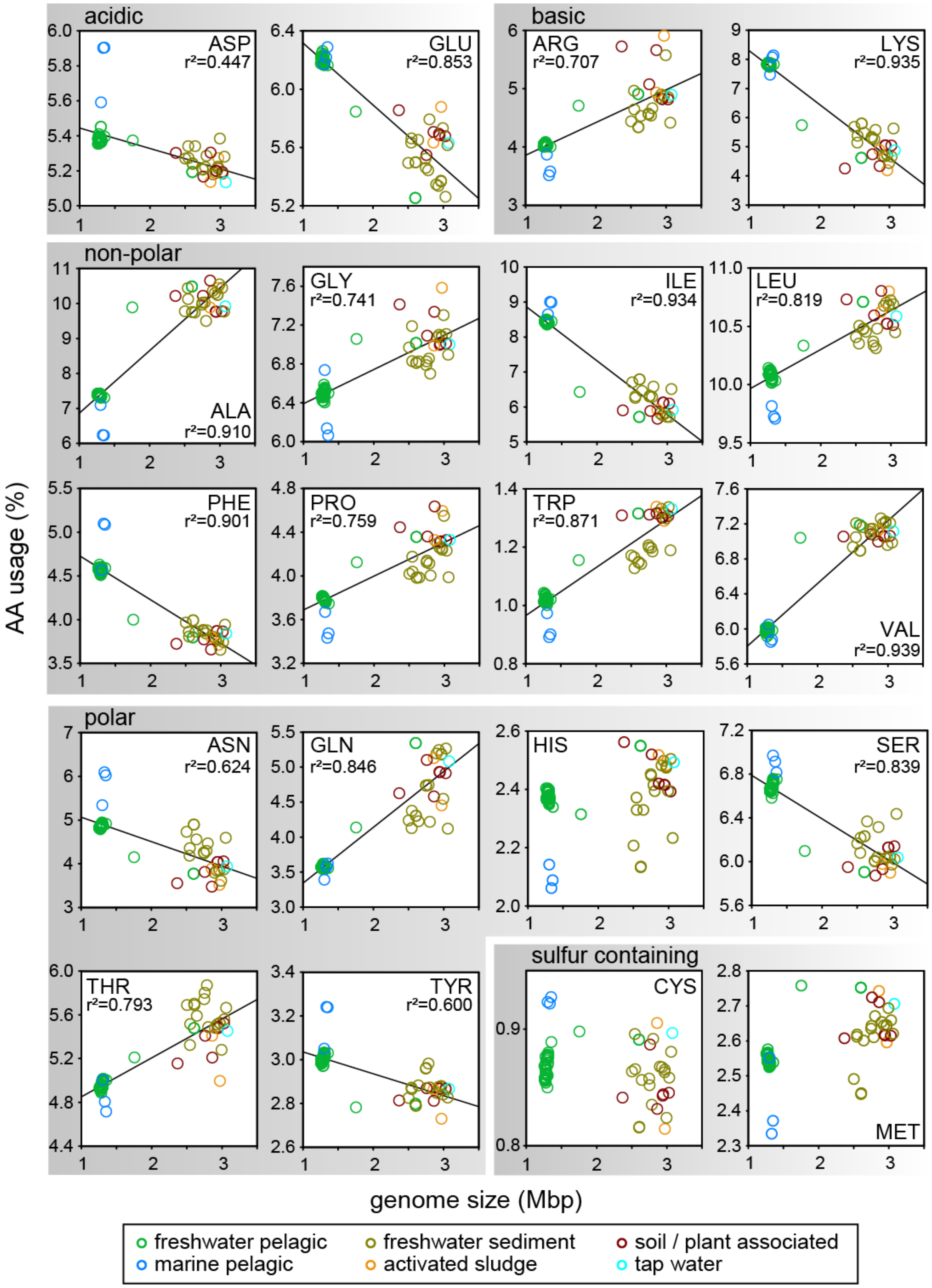

Figure S6: Amino acid usage of Methylophilaceae in relation to genome size.

a

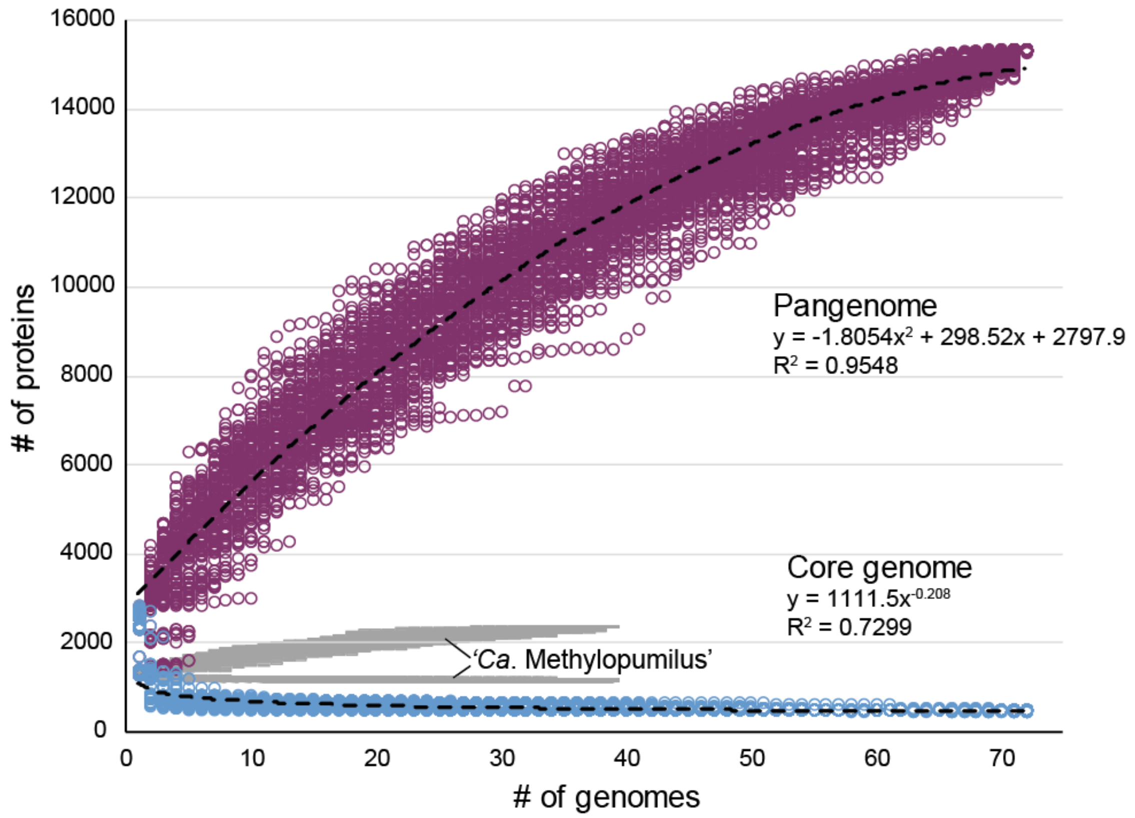

b

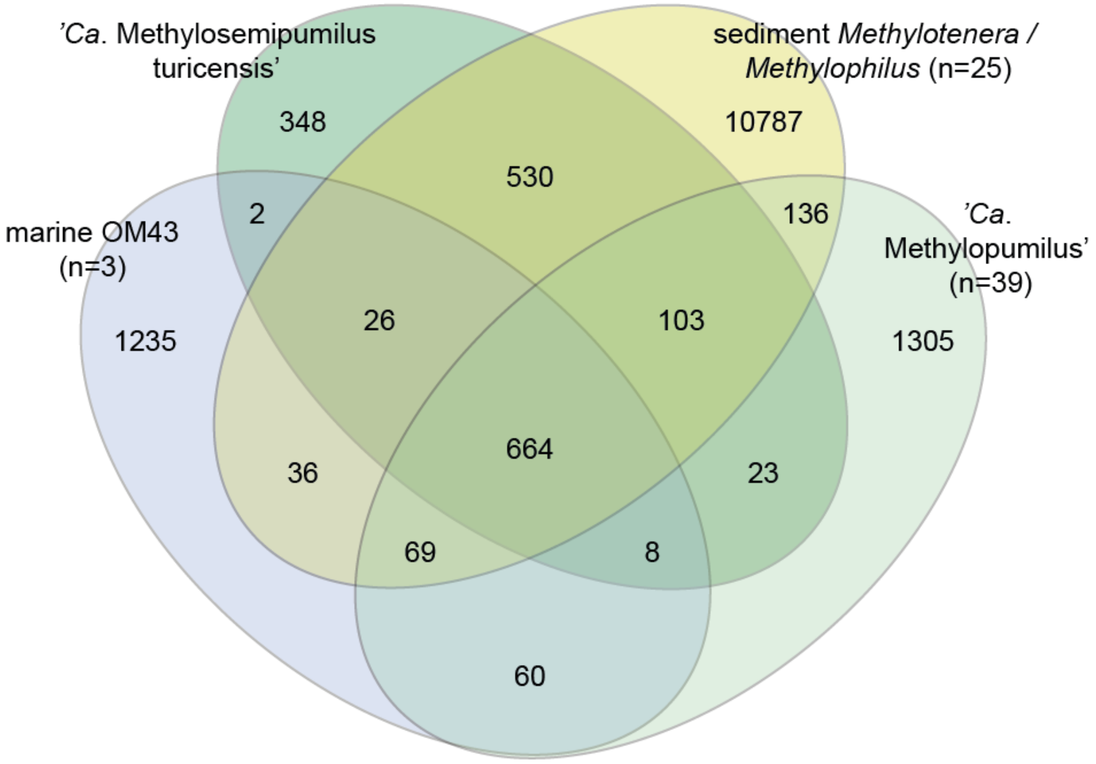

Figure S7: Core- and pangenome analysis of Methylophilaceae.

(a) Core- and pangenome analysis of all 72 Methylophilaceae strains in relation to 39 '*Ca*. Methylopumilus spp. strains. (b) Shared protein families of sediment *Methylophilus* and *Methylotenera*, '*Ca*. Methylosemipumilus turicensis', '*Ca*. Methylopumilus spp.' and marine OM43 at a 50% identity clustering.

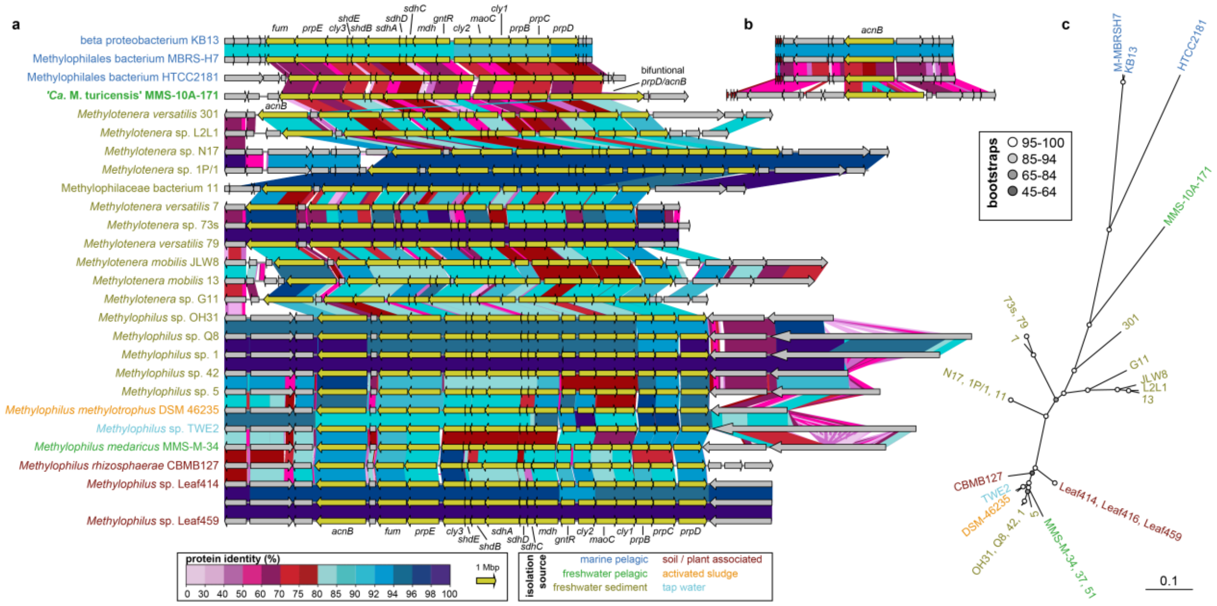

Figure S8: Methylcitric acid (MCA) pathway

(a) Arrangement and protein similarities of genes coding for the methylcitric acid (MCA) pathway marked in dark yellow and flanking genes. (b) Similarities of *acnB* genes in marine OM43 and 'Ca. M. turicensis', located at different genomic regions. (c) RAxML tree (100 bootstraps) of concatenated MCA proteins.

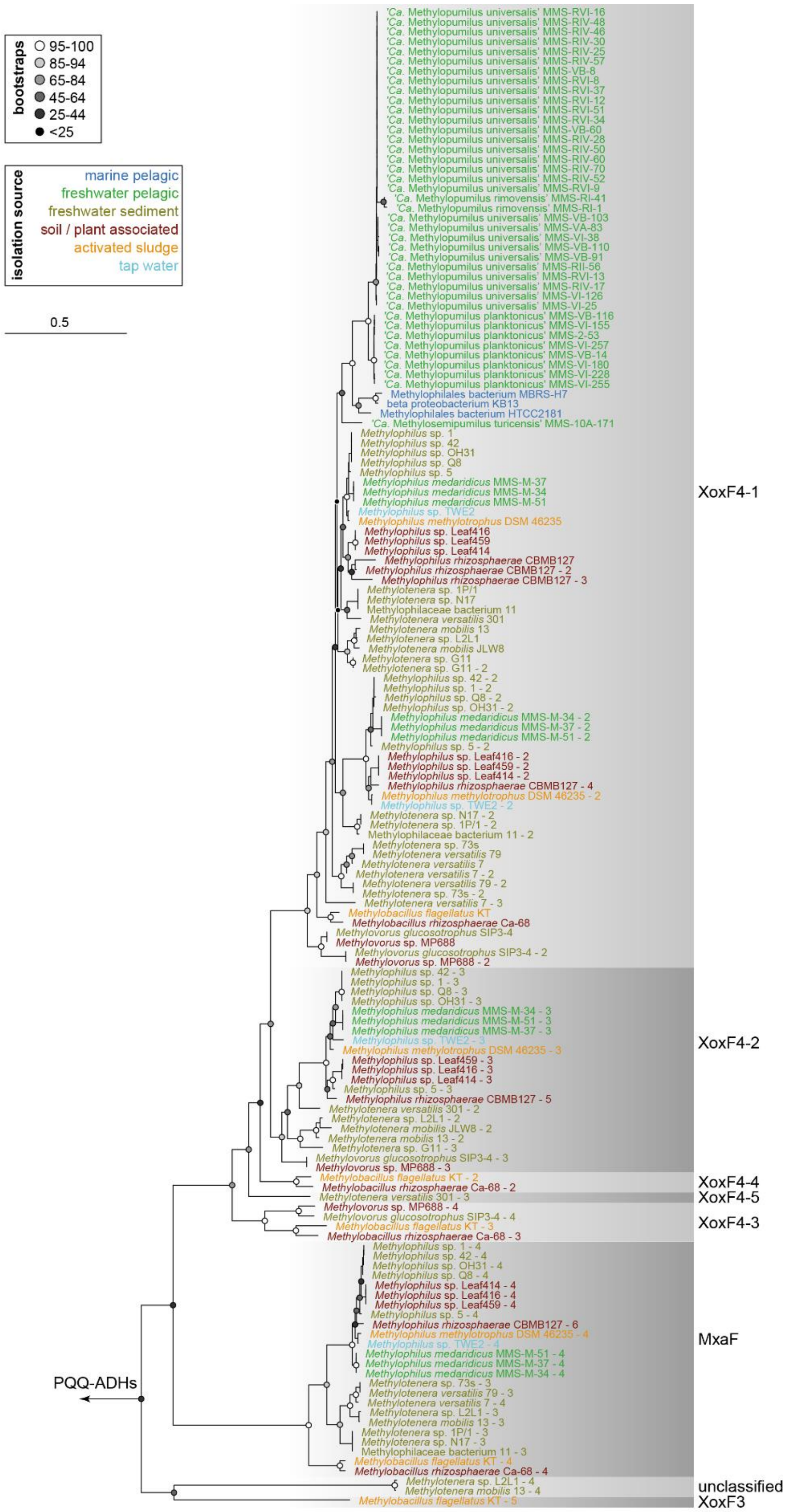

Figure S9: XoxF and MxaF type methanol dehydrogenases

Phylogenetic tree (100 bootstraps) of XoxF and MxaF type methanol dehydrogenases in Methylophilaceae. Isolation sources of strains are indicated by different colours. Note that some strains have multiple copies of XoxF and MxaF. Several PQQ-dependent alcohol dehydrogenases (PQQ-ADHs type 9) and other distantly related dehydrogenases were used as outgroup.

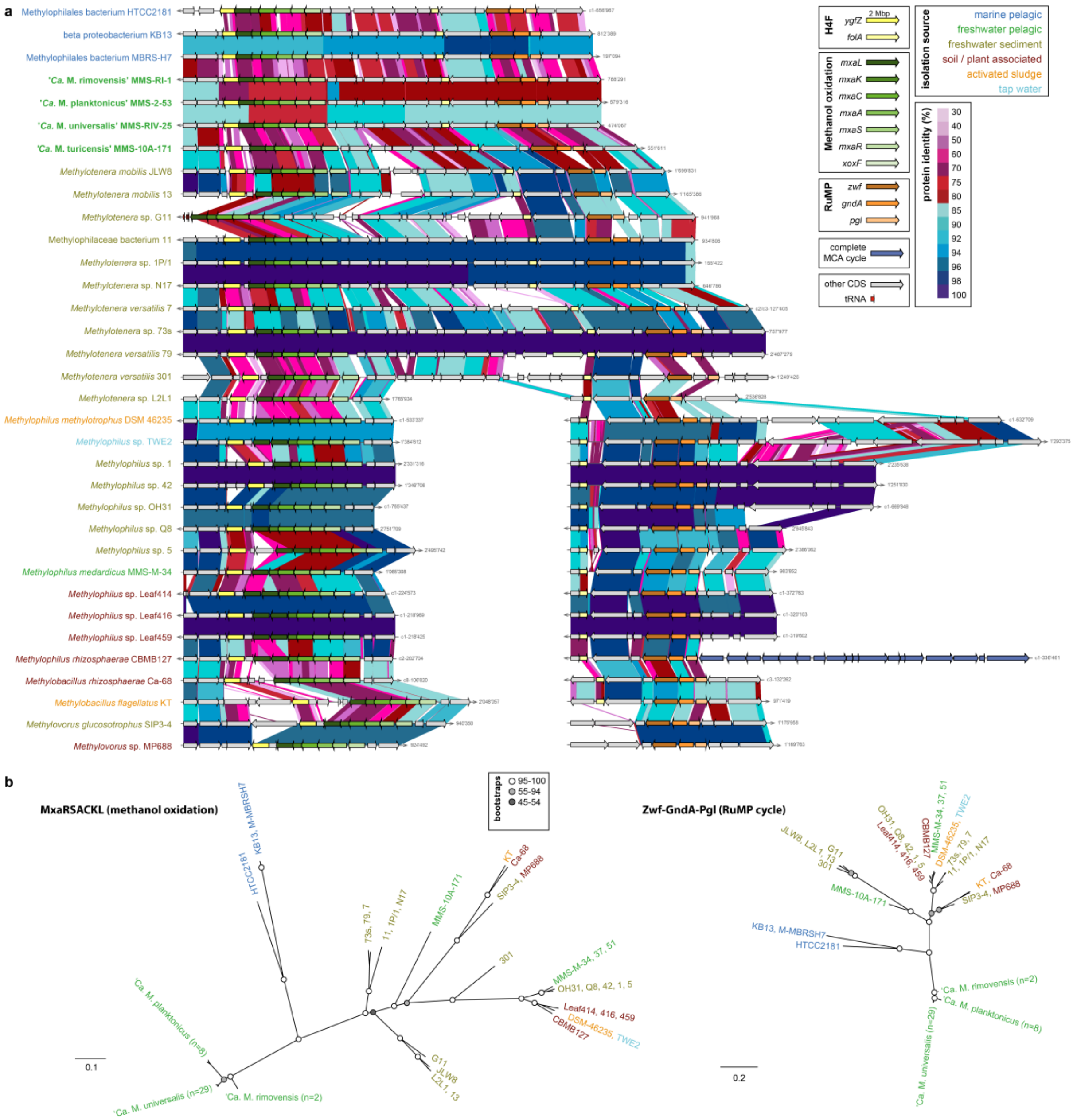

Figure S10: Conserved methylotrophic pathways - H4F, methanol oxidation and RuMP

(a) Synteny of different methylotrophic pathways in Methylophilaceae. Genes involved in methanol oxidation, the H4F-pathway of formaldehyde oxidation, the RuMP cycle, and a complete MCA cycle are marked by different colours, as well as protein similarities (%) of individual genes. (b) Phylogenetic trees (100 bootstraps) of concatenated MxaRSACKL (left) and Zwf-GndA-Pgl proteins (right). Isolation sources of strains are indicated by different colours.

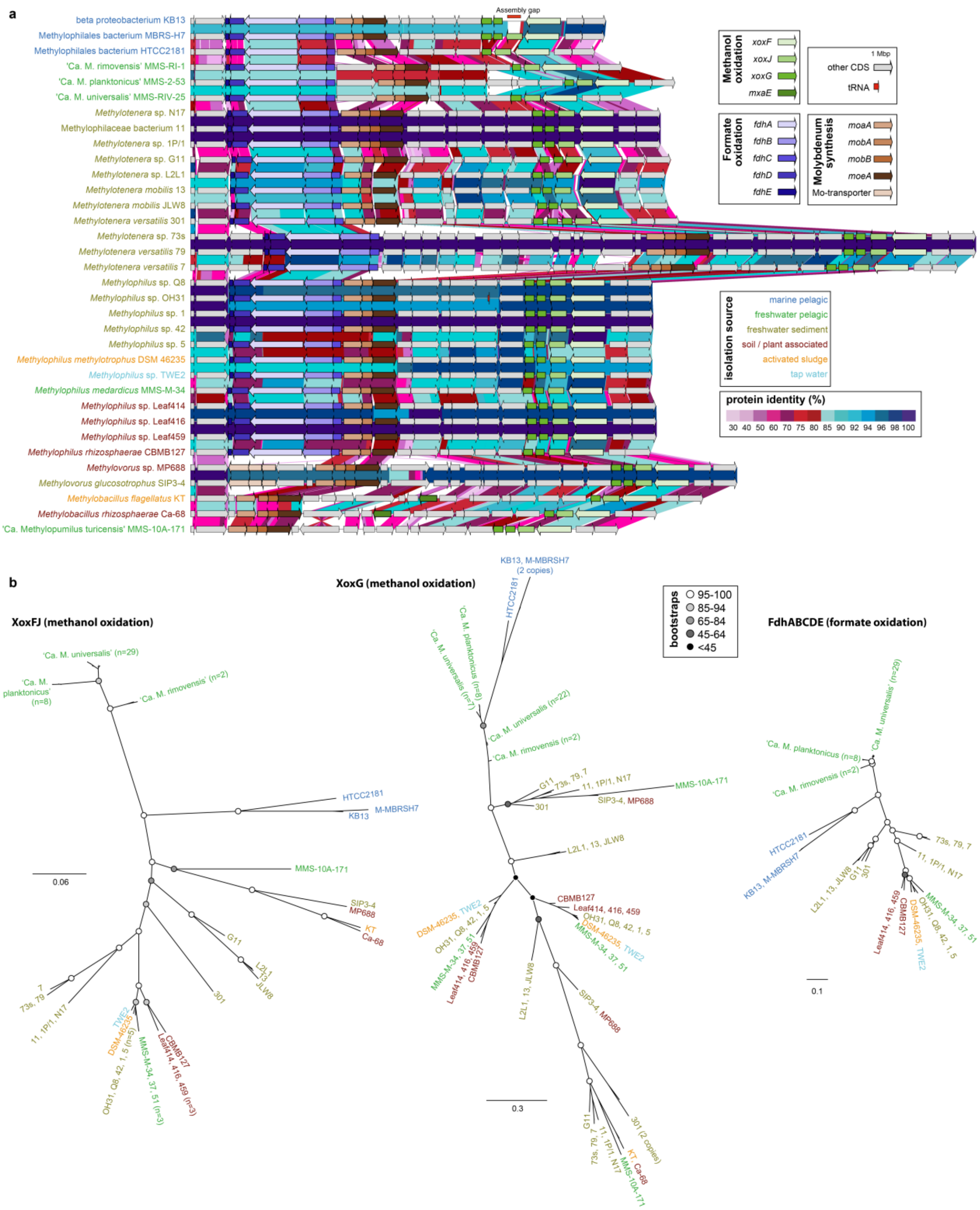

Figure S11: Conserved methylotrophic pathways - methanol and formate oxidation

(a) Synteny of different methylotrophic pathways in Methylophilaceae. Genes involved in methanol oxidation, formate oxidation and synthesis of the cofactor molybdenum are marked by different colours, as well as protein similarities (%) of individual genes. (b) Phylogenetic trees (100 bootstraps) of concatenated XoxFJ (left), XoxG (middle) and FdhABCDE (right). Isolation sources of strains are indicated by different colours. Note that some strains have multiple copies of XoxG.

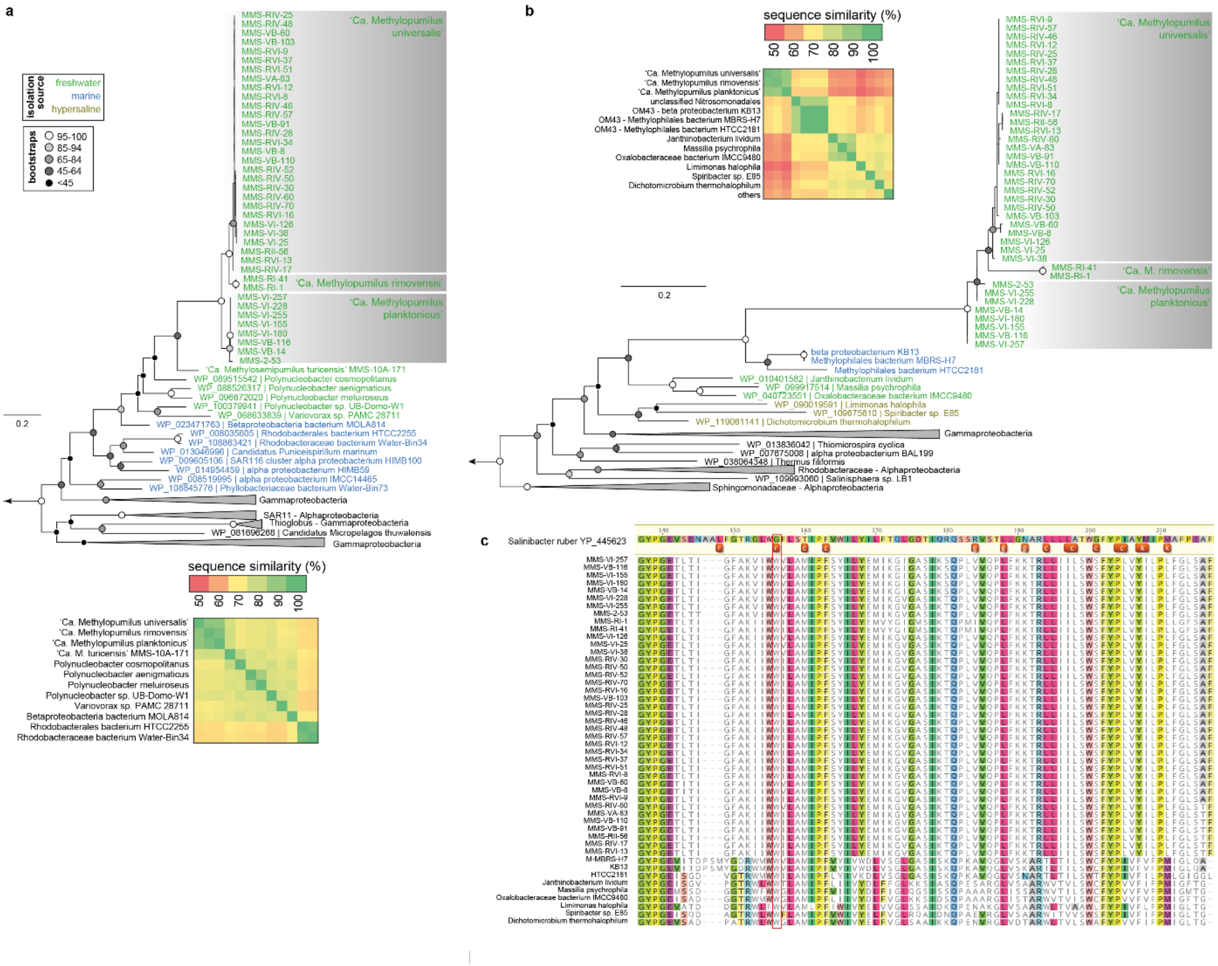

Figure S12: Horizontal gene transfers of different rhodopsins

RAxML trees (100 bootstraps) of (a) proteorohodopsins of '*Ca*. Methylopumilus spp.' and '*Ca*. Methylosemipumilus turicensis' and (b) xantho-like rhodopsins of '*Ca*. Methylopumilus spp.' and marine OM43. Protein sequence identities for the closest relatives are given below (a) or at the left side (b). Isolation sources of the closest relatives are indicated by different colours, actinorhodopsins were used as outgroup for both trees. (c) Amino acid alignment of xantho-like rhodopsins with residues involved in caroteinoid binding in *Salinibacter ruber* DSM13855 (Luecke et al. 2008, Imasheva et al. 2009). Letters c, g, k, and r stand for the chain, glucoside, keto group, and ring, respectively, and indicate part of the carotenoid in the vicinity of a residue. Note ring binding site at position 156 highlighted in red, where glycine is replaced with tryptophan in all strains indicating that the antenna might not bind.

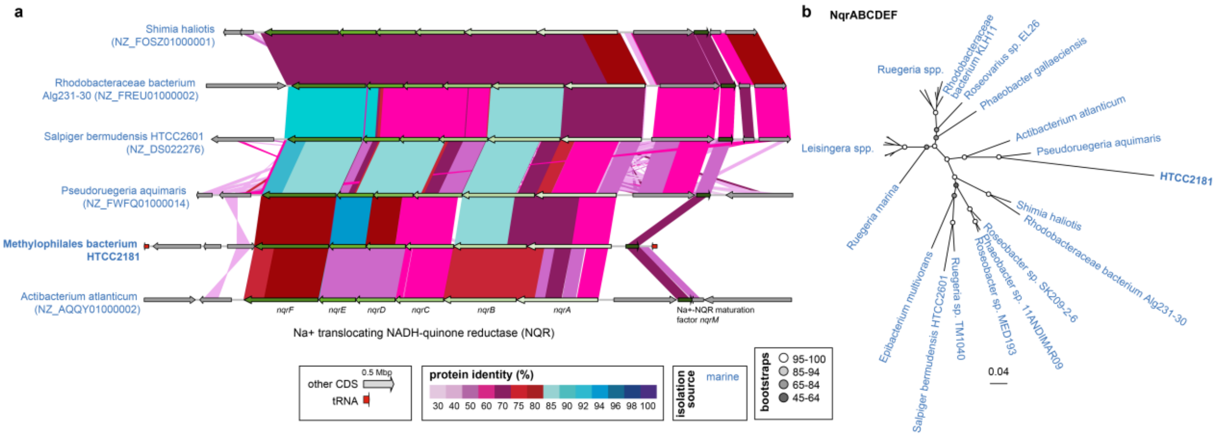

Figure S13: Horizontal gene transfer of the NQR pathway in marine HTCC2181

(a) Arrangement and protein similarities of genes coding for the Na+ translocating NADH-quinone reductase (NQR) marked in green colours and flanking genes. (b) RAxML tree (100 bootstraps) of concatenated NqrABCDEF proteins of the NQR pathway. Isolation sources of the strains are indicated by different colours.

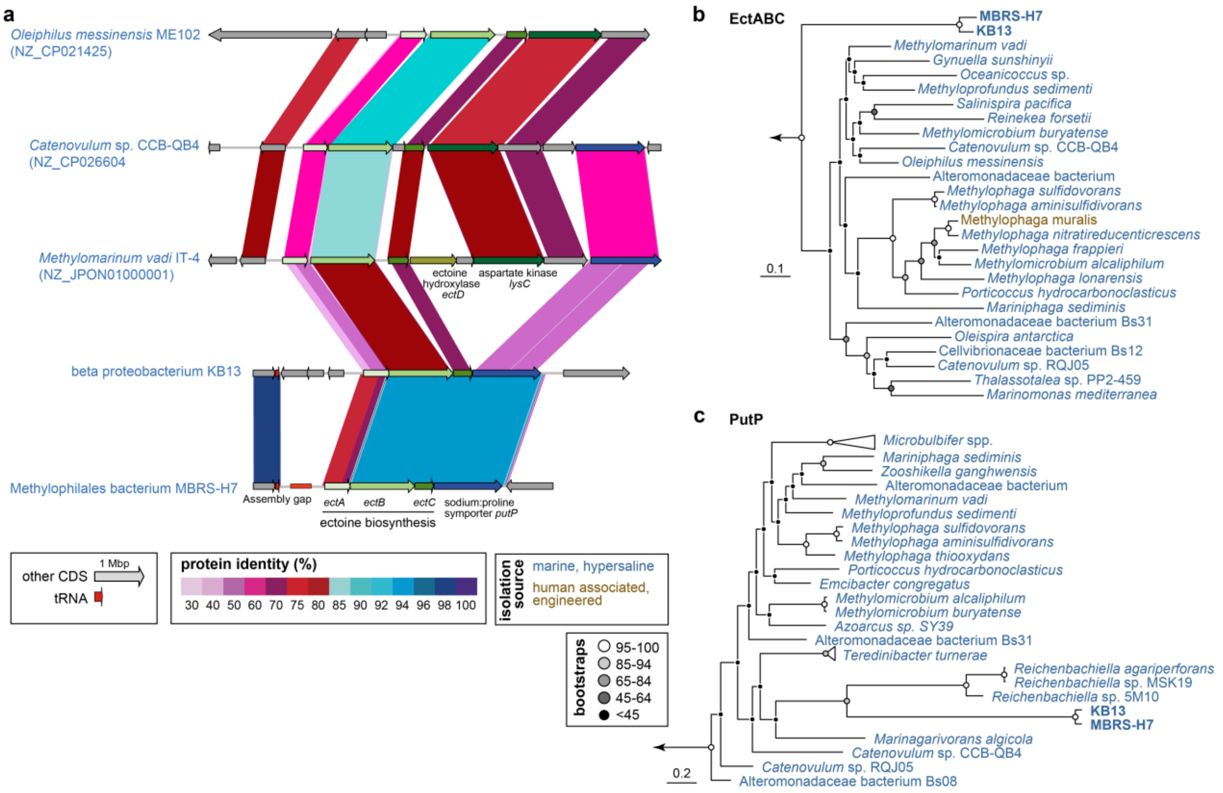

Figure S14: Horizontal gene transfer of an ectoine biosynthesis pathway and a sodium:proline symporter in marine OM43

(a) Arrangement and protein similarities of genes coding for the ectoine biosynthesis pathway (*ectABC*) marked in green colours and flanking genes, including a sodium:proline symporter (marked in blue). Aspartate kinase *lysC* as well as aspartate-semialdehyde dehydrogenase *asd* are located elsewhere in the genomes of KB13 and MBRS-H7 and more similar to *lysC* and *asd* of other Methylophilaceae. RAxML trees (100 bootstraps) of (b) concatenated EctABC proteins of the ectoine biosynthesis pathway and (c) the sodium:proline symporter PutP. Isolation sources of the strains are indicated by different colours.

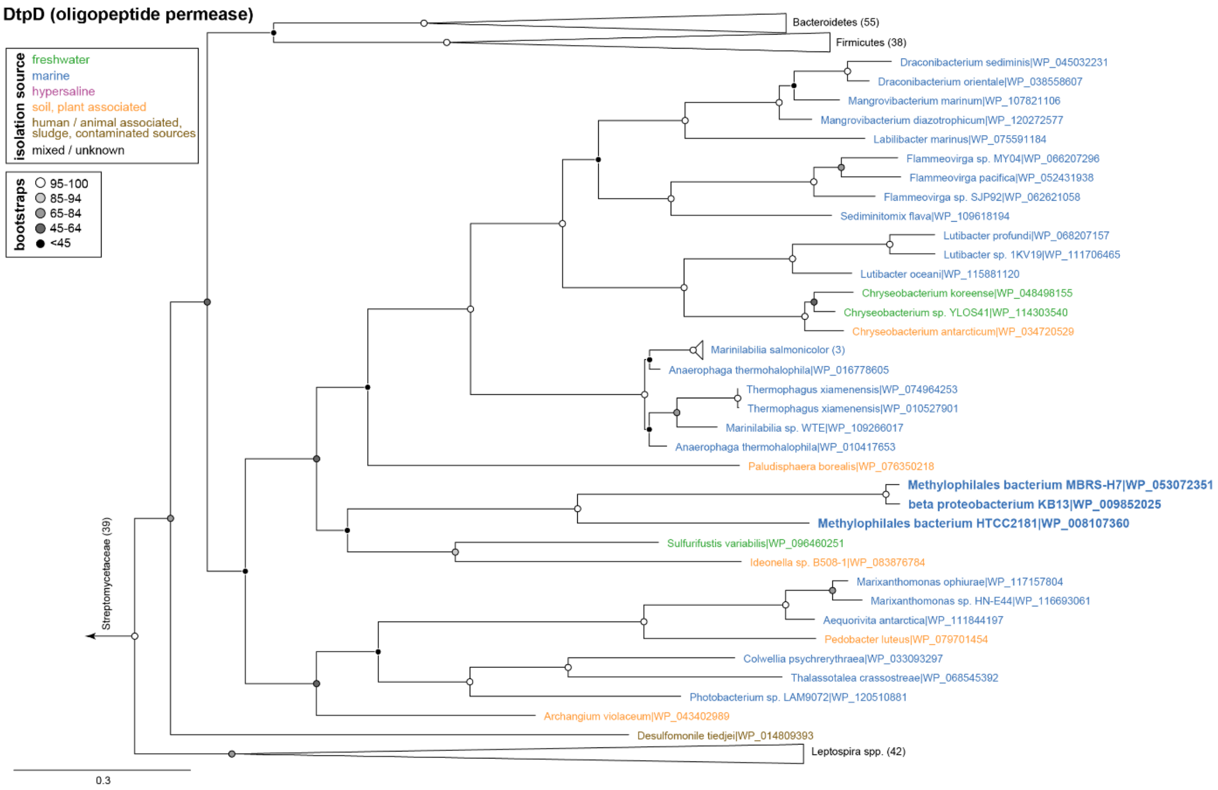

Figure S15: Horizontal gene transfer an oligopeptide permease in marine OM43

RAxML trees (100 bootstraps) of the dipeptide/tripeptide permease DtpD unique for the marine OM43 cluster (in bold). Isolation sources of the closest relatives are indicated in different colours.

a) AlsT (sodium:alanine symporter)

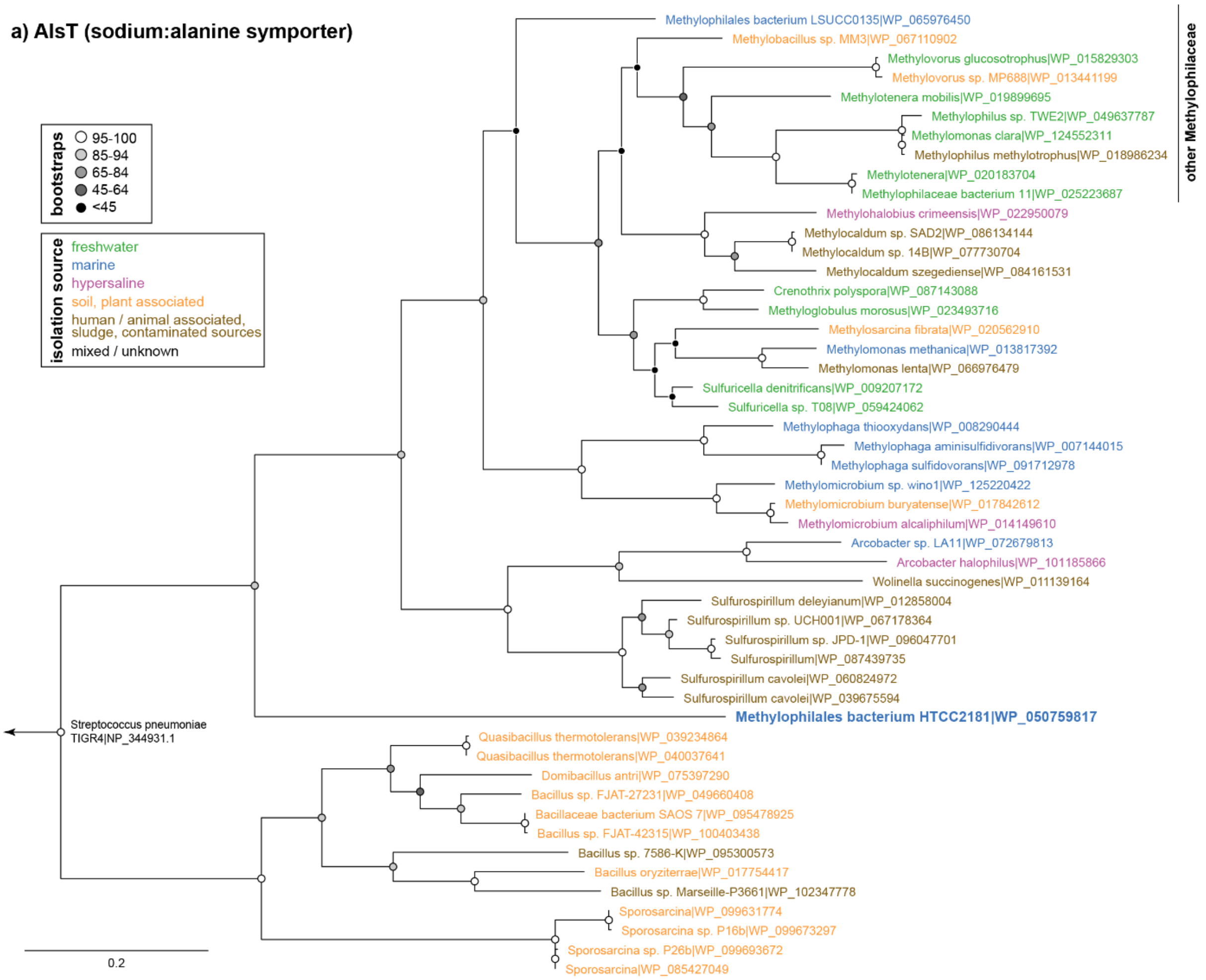

b) ActP (sodium:acetate symporter)

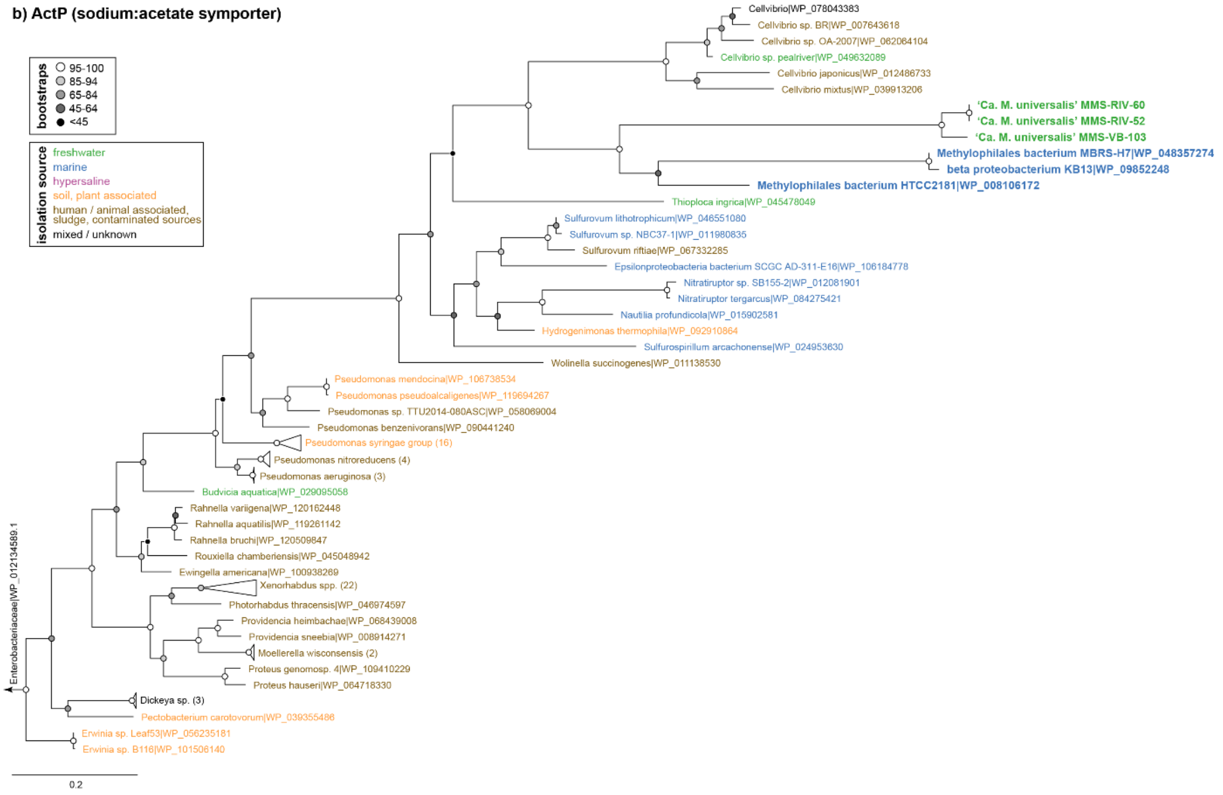

c) GltT (sodium:dicarboxylate symporter)

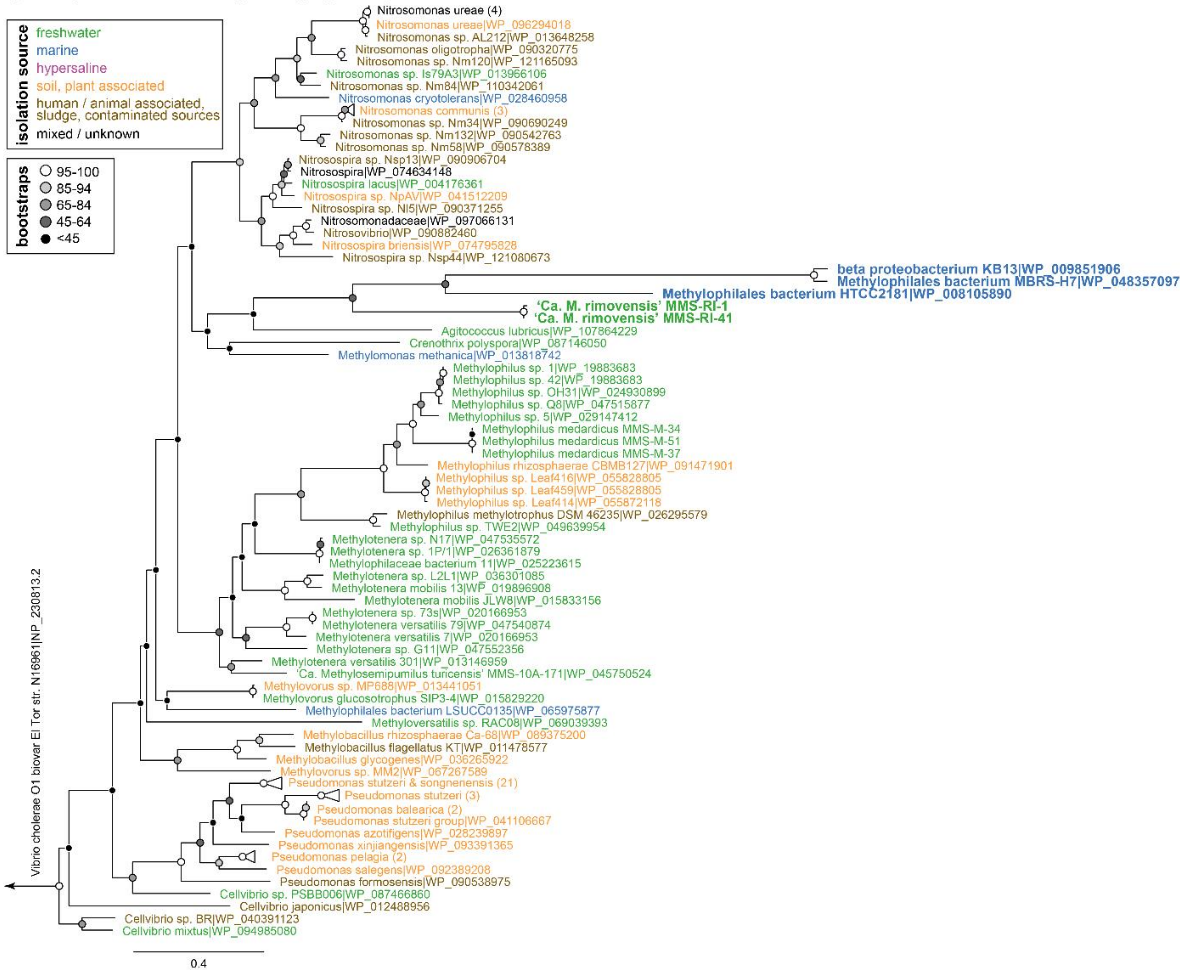

d) NhaP2 (sodium:proton antiporter)

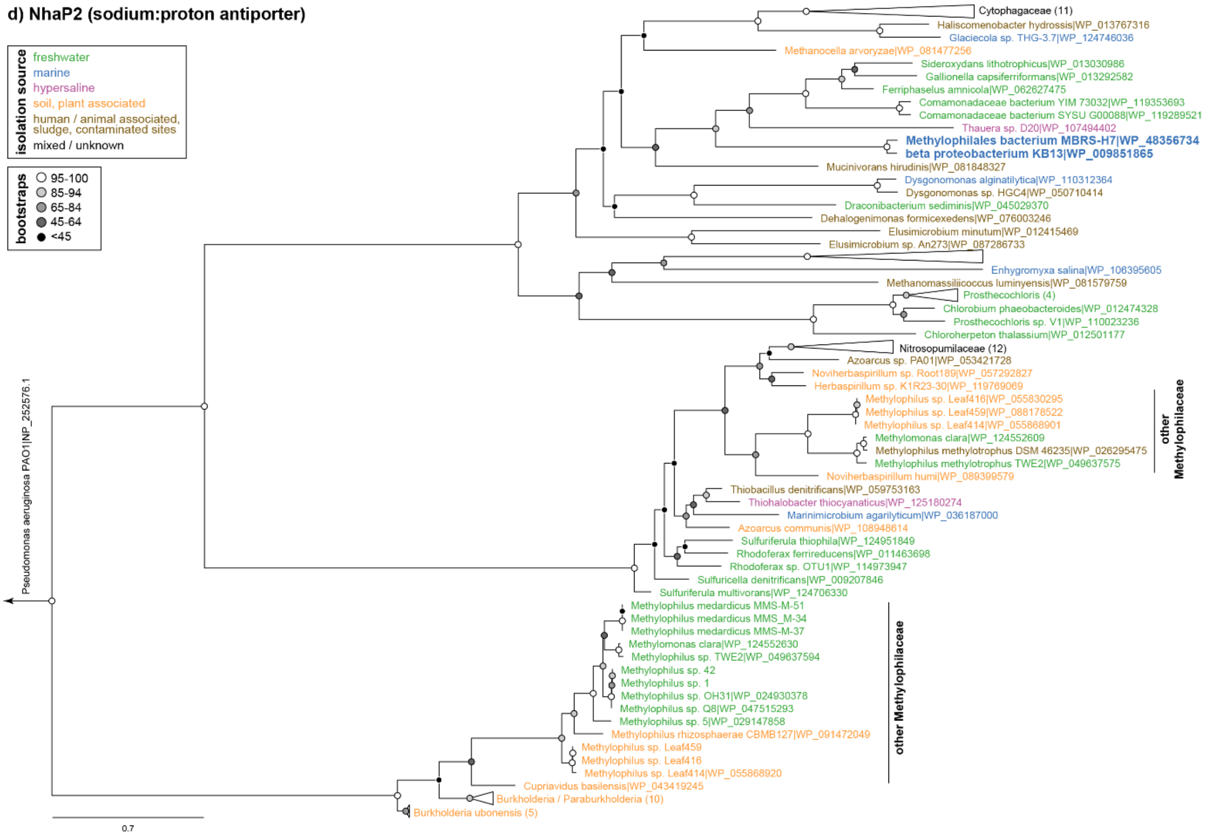

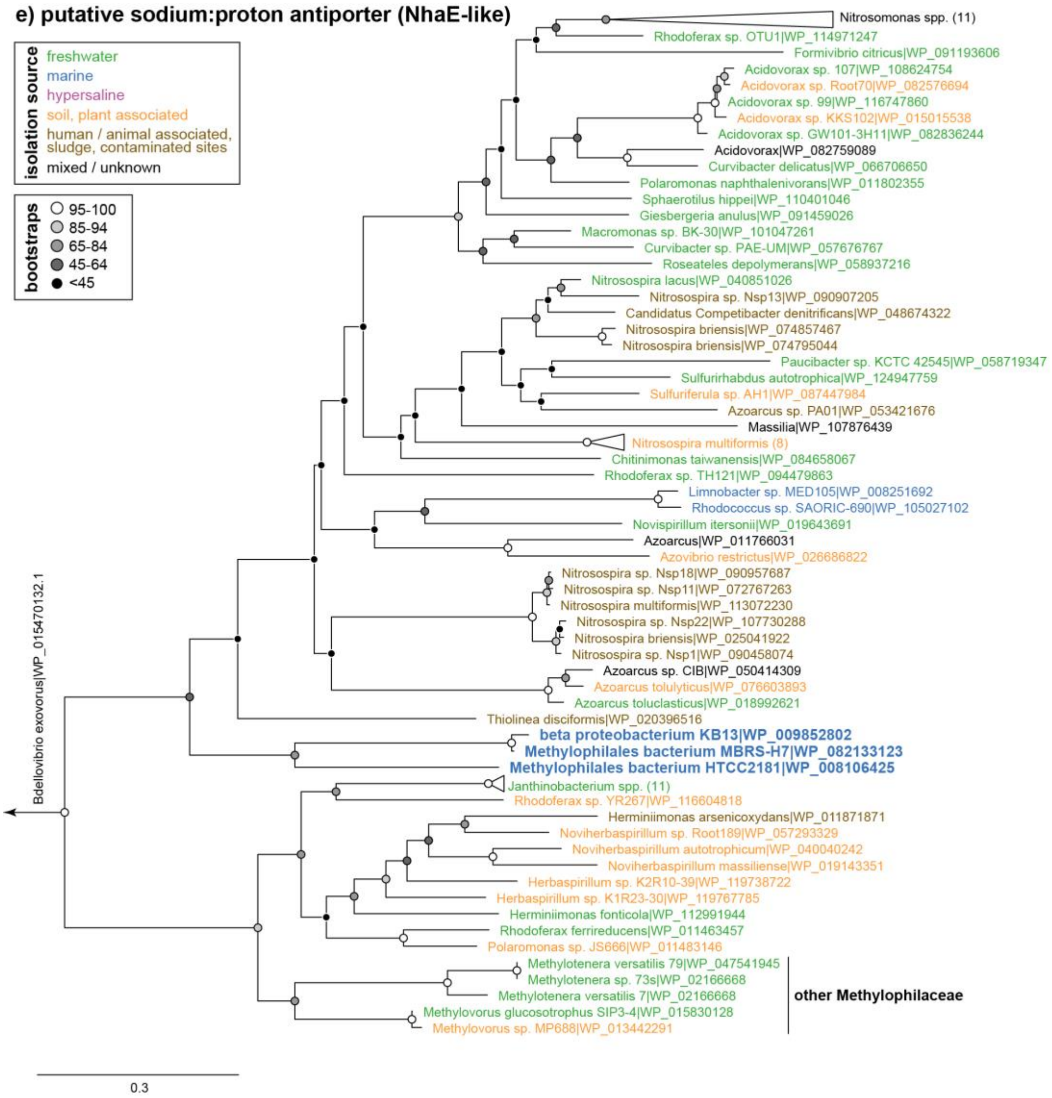

Figure S16: Horizontal gene transfer of sodium transporters in marine OM43

RAxML trees (100 bootstraps) of (a) the sodium:alanine symporter AlsT, the sodium:acetate symporter ActP, (c) the sodium:dicarboxylate symporter GltT, (d) the sodium:proton antiporter NhaP2, and (e) another putative sodium:proton antiporter (NhaE-like). Marine OM43 and ‘*Ca*. Methylopumilus‘ strains are highlighted in bold, isolation sources of the closest relatives are indicated in different colours.
